# Supplementary material for: Observational evidence for on-shelf heat transport driven by dense water export in the Weddell Sea
Source: Nat Commun. 2023 Mar 7;14:1022. doi: 10.1038/s41467-023-36580-3 (PMC9992355; doi:10.1038/s41467-023-36580-3)
Supplement: Supplementary file 1 — Supplementary Information [file 41467_2023_36580_MOESM1_ESM.pdf]

Supplementary information

Elin Darelius[1*], Kjersti Daae[1†], Vår Dundas[1†], Ilker Fer[1†], Hartmut H. Hellmer[2†], Markus Janout[2†], Keith W. Nicholls[3†], Jean-Baptiste Sallée[4†] and Svein Østerhus[5†]

[1]Geophysical Institute, University of Bergen and the Bjerknes Centre for climate Research, Alleg. 70, Bergen, Norway.
[2]Alfred Wegener Institute, Helmholtz Centre for Polar and Marine Research, Am Handelshafen 12, Bremerhaven, Germany.
[3] British Antarctic Survey, High Cross, Madingley Road, Cambridge, UK.
[4]Sorbonne Université, CNRS, LOCEAN, 4, Place Jussieu, Paris, France.
[5]NORCE Norwegian Research Centre AS and the Bjerknes Centre for Climate Research, Jahnebakken 5, Bergen, Norway.

*Corresponding author(s). E-mail(s): elin.darelius@uib.no;
Contributing authors: kjersti.daae@uib.no; var.dundas@uib.no; ilker.fer@uib.no; hartmut.hellmer@awi.de; markus.janout@awi.de; kwni@bas.ac.uk; jean-baptiste.sallee@locean.ipsl.fr; svos@norceresearch.no;
†These authors contributed equally to this work.

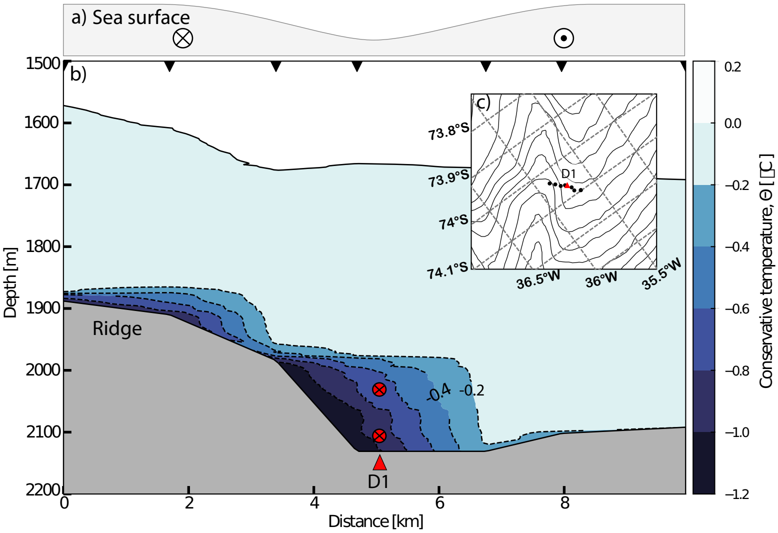

Supplementary Figure 1: **Temperature section across the ridge.** a) Sketch showing the Sea Surface Height (SSH) – signature suggested by Morrison et al [1] to develop above a topographically steered downslope flow such as the one shown in (b). Note that SSH observations from the (normally ice-covered) study region do not exist. b) Conservative Temperature section across the ridge downstream of the Filchner Trough [2], where the low temperatures identify the dense plume water. The position of the mooring D1 (See Fig. 1 and Table 1 in the main article for location and details on the mooring deployment) and the direction of the observed currents are indicated in red.

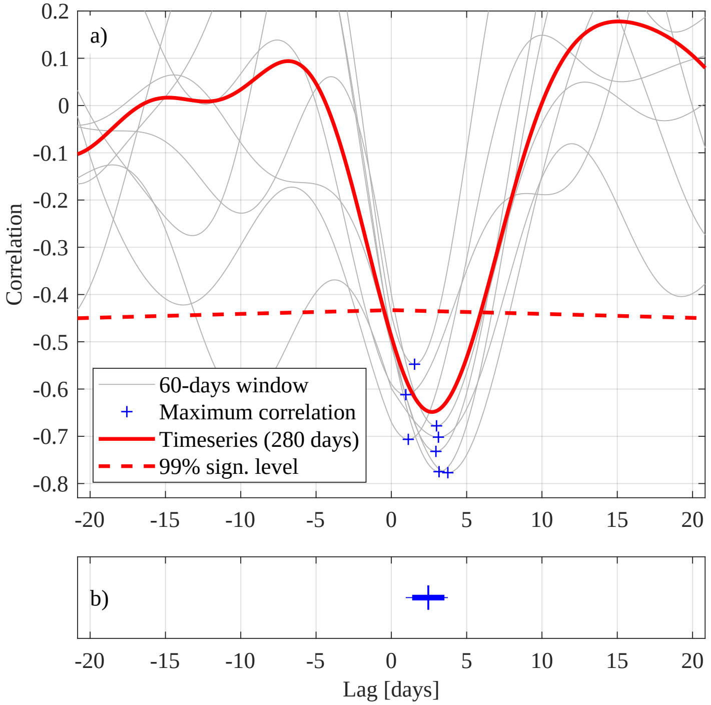

Supplementary Figure 2: **Lagged correlation of velocity time series from mooring D1 and D2** a) Correlation as a function of lag between the across slope velocity component at D1 and D2, where positive lag indicates that D2 leads. The red line shows the correlation for the complete filtered time series (280 days), and thin grey lines show the correlation calculated for 60 days long, 50% overlapping subsections of the filtered time series. The maximum correlation for each subsection is highlighted with a blue cross, and the 99% significance level calculated following Sciremammano [3] for the filtered time series is shown with a dashed red line. b) The box plot shows the range (thin blue line), the mean (vertical bar), and the standard deviation (thick blue line) of the lags obtained from the subsections in (a).

Supplementary References

[1] Morrison, A. K., McC. Hogg, A., England, M. H. & Spence, P. Warm Circumpolar Deep Water transport toward Antarctica driven by local dense water export in canyons. *Science Advances* **6** (18), 1–10 (2020). https://doi.org/10.1126/sciadv.aav2516 .

[2] Darelius, E. & Wåhlin, A. K. Downward flow of dense water leaning on a submarine ridge. *Deep-Sea Research Part I-Oceanographic Research Papers* **54** (7), 1173–1188 (2007). https://doi.org/10.1016/j.dsr.2007.04.007 .

[3] Sciremammano, F. J. A Suggestion for the Presentation of Correlations and Their Significance Levels. *Journal Of Physical Oceanography* **9**, 1273–1276 (1979) .
